# Association of Serum Albumin with Markers of Nutritional Status among HIV-Infected and Uninfected Rwandan Women

**DOI:** 10.1371/journal.pone.0035079

**Published:** 2012-04-19

**Authors:** Jean-Claude Dusingize, Donald R. Hoover, Qiuhu Shi, Eugene Mutimura, Elizabeth Kiefer, Mardge Cohen, Kathryn Anastos

**Affiliations:** 1 Albert Einstein College of Medicine, Bronx, New York, United States of America; 2 Department of Statistics and Biostatistics and Institute for Health, Health Care Policy and Aging Research, Rutgers University, Piscataway, New Jersey, United States of America; 3 New York Medical College, Valhalla, New York, United States of America; 4 Women's Equity in Access to Care and Treatment (WE-ACTx) and Kigali Health Institute, Kigali, Rwanda; 5 Stroger (Cook County) Hospital and Rush University, Chicago, Illinois, United States of America; National Taiwan University Hospital, Taiwan

## Abstract

**Introduction:**

The objectives of this study are to address if and how albumin can be used as an indication of malnutrition in HIV infected and uninfected Africans.

**Methods:**

In 2005, 710 HIV-infected and 226 HIV-uninfected women enrolled in a cohort study. Clinical/demographic parameters, CD4 count, albumin, liver transaminases; anthropometric measurements and Bioelectrical Impedance Analysis (BIA) were performed. Malnutrition outcomes were defined as body mass index (BMI), Fat-free mass index (FFMI) and Fat mass index (FMI). Separate linear predictive models including albumin were fit to these outcomes in HIV negative and HIV positive women by CD4 strata (CD4>350,200–350 and <200 cells/µl).

**Results:**

In unadjusted models for each outcome in HIV-negative and HIV positive women with CD4>350 cells/µl, serum albumin was not significantly associated with BMI, FFMI or FMI. Albumin was significantly associated with all three outcomes (p<0.05) in HIV+ women with CD4 200–350 cells/µl, and highly significant in HIV+ women with CD4<200 cells/µl (P<0.001). In multivariable linear regression, albumin remained associated with FFMI in women with CD4 count<200 cells/µl (p<0.01) but not in HIV+ women with CD4>200.

**Discussion:**

While serum albumin is widely used to indicate nutritional status it did not consistently predict malnutrition outcomes in HIV- women or HIV+ women with higher CD4. This result suggests that albumin may measure end stage disease as well as malnutrition and should not be used as a proxy for nutritional status without further study of its association with validated measures.

## Introduction

Sub-Saharan Africa is by far the region most-affected by the AIDS epidemic [1]. The region has just over 10% of the world's population, but is home to 68% of all people living with HIV [1]. HIV infection has adverse consequences on economic development and well being including household food insecurity [2]. In Rwanda HIV prevalence is approximately 3% and is higher in women than in men (3.6 vs 2.3% respectively) [3]. As in all of sub-Saharan Africa, HIV infection in Rwanda has adverse consequences on economic development and well being including household food insecurity. This situation is aggravated by poverty, which affects 60% of Rwandans, and a high prevalence (>30%) of malnutrition regardless of HIV status [3].

Africans with advanced HIV disease may also experience wasting and loss of fat-free mass secondary to several factors. Oral and gastrointestinal infections associated with HIV can make food difficult to ingest; other manifestations of advanced HIV disease can also interfere with an individual's ability to ingest nutrients. Additionally, elevated proinflammatory cytokines in untreated HIV infection, including interleukin-1, interleukin-6, and tumor necrosis factor α [4], [5] can cause anorexia or may promote catabolism causing weight loss despite sufficient intake of energy and protein [4], [6].

Considering the combined burden of malnutrition in sub Saharan Africa, and the catabolic condition found in HIV positive patients, it is important to be able to measure malnutrition in order to determine if malnutrition is associated with adverse outcomes in HIV infected Africans. To that end, body composition is an important measure of nutritional status. The tool most commonly used to assess adult malnutrition is weight adjusted for height, expressed as “body mass index” (BMI): weight in kilograms divided by the square of height measured in meters. The World Health Organization classifies a BMI<18.5 as a sign of malnutrition [7]. However BMI can be insensitive to changes in lean muscle mass (LMM), which is a more accurate marker of recent change in nutritional status [8]. In women, who represent >60% of the African HIV infected population, LMM is lower and proportion of total body fat is higher than in men.

Bioelectrical impedance analysis (BIA) measures body composition, in particular, fat free mass, which enables estimation of LMM. BIA is a good tool for assessing body cell mass loss in HIV positive patients and compares favorably with gold standard methods, for example dual energy x-ray absorptiometry, (DXA)(r = 0.9) [9], [10]. Because of the limitations in expressing body composition data as kilograms (eg, kg of fat-free mass or fat mass), similar to BMI it has been suggested to use height(ht) normalized indices: the fat mass index (FMI, kg of fat mass/ht-m^2^) and the fat-free mass index (FFMI, kg of fat-free mass/ht-m^2^) as indicators of nutritional status in patients [11], [12].

Laboratory parameters may provide an alternate way to measure malnutrition which may reflect a more immediate state of malnutrition than mass indices which have built up over time. History, physical examination, and anthropometric measurements are essential parts of nutritional evaluation [13]. However, these tools can be highly subjective and rely on the knowledge and experience of the evaluator. Incorporating biochemical measurements into routine nutritional assessment provides an often-needed objective dimension. Serum albumin, which is routinely measured in developed countries and available in most inpatient and many ambulatory settings in Africa, is the most used laboratory parameter for nutritional assessment with low serum albumin indicating malnutrition [14], [15]. However, its ability to discriminate fully between patients with normal and abnormal nutritional status is not yet well studied. Low serum albumin has also been found to occur with a variety of acute and chronic diseases and thus is associated with higher mortality in a number of different populations in pathways that may not solely result from malnutrition. For this reason serum albumin is also often used as a prognostic indicator of disease progression [16], [17]. Several studies have indicated that lower concentrations of serum albumin, even within the “normal” range, in HIV infected persons are associated with more rapid progression to AIDS and all-cause mortality, independent of other prognostic indicators and treatments including CD4 cell count, HIV-1 viral load, and highly active antiretroviral therapy (HAART) [18], [19].

An important question in treating HIV-infection in Africa is the effect of comorbid malnutrition on response to HAART [20]. To this end it may be important to know if albumin can be used as an immediate indication of malnutrition, in both research and clinical settings. Despite the use of serum albumin level as a standard indicator of nutritional status, its sensitivity and specificity for malnutrition are not well established in HIV-infected individuals where low serum albumin often results (independently of malnutrition) from advanced HIV infection. The present cross-sectional study uses data collected at entry into a cohort study conducted in Kigali, Rwanda, to assess the association of serum albumin with other markers of nutritional status and whether serum albumin can be used for nutritional assessment among antiretroviral naïve HIV infected and HIV uninfected patients.

## Materials and Methods

### Study population

The Rwandan Women's Interassociation Study and Assessment (RWISA) is an observational cohort study designed to assess the effectiveness and toxicity of antiretroviral therapy in HIV-infected Rwandan women. Written informed consent was obtained in accordance with protocols approved by the Rwandan National Ethics Committee and the Institutional

Review Board of Montefiore Medical Center, Bronx NY, USA. In 2005, 710 HIV-infected and 226 HIV-uninfected women enrolled with follow-up visits occurring every six months. Participants were recruited through grassroots women's organizations and clinical care sites for HIV-infected patients. Inclusion criteria were: age at least 25 years at study entry, willingness to give informed consent, and no history of receiving antiretroviral treatment except for possibly single dose nevirapine to prevent mother-to-child transmission of HIV.

### Anthropometrics and body composition measurements

Anthropometric measurements (height and weight, skin-fold thickness, body circumference at the waist, hip, and chest) and Bioelectrical Impedance Analysis (BIA) were performed. BIA is a simple, noninvasive technique that has been recommended for nutritional assessment studies in the clinical setting [21] and has been shown to be sufficiently precise for clinical investigation of body composition analysis [21], [22].These evaluations were undertaken by nurses trained in proper anthropometry with anatomic placement of the tape measure and location of skin folds by clinical trainers. Anthropometric measurements were made in duplicate and in the case of discrepancy a third measurement was done. Standing height and weight were measured while the participant was wearing light clothing and no shoes. Two measurements of skin folds at mid triceps, front thigh, subscapular, and suprailiac were taken and read to the nearest 0.2 mm, and an average obtained for each site. Impedance measurements were taken in duplicate using a standard tetrapolar electrode placement on the hand and foot. Total body fat and fat-free mass (FFM) were calculated from BIA measurements using gender-specific equations that were previously cross-validated in a sample of patients (white, black, and Hispanic) with and without HIV infection [23] and have been applied elsewhere in African studies [24], [25]

Body mass index was calculated as weight(kg)/(height^2^(m). The Fat Free Mass Index (FFMI) was obtained by dividing calculated fat-free mass by height-squared. Fat Mass Index (FMI) was calculated by BMI minus FFMI. In some participants with very low BMI (<17.0) the calculated FFM was greater than the weight; in these cases the FFM was set to equal the weight and FMI to zero. Sum of skin folds variable was generated by adding together thigh, triceps, and subscapular skin folds.

### Patient history and clinical parameters

At study entry, participants were interviewed and a physical examination was conducted. Income was reported as Rwandan francs (RWF) monthly, and is shown in RWF and US dollars. Stage IV HIV disease WHO illness was determined from participant self-report of extrapulmonary tuberculosis, HIV wasting syndrome, pneumocystis pneumonia, recurrent severe pneumonia, Kaposi's sarcoma, chronic herpes simplex infection of more than one month's duration, esophageal candidiasis, HIV encephalopathy, extra pulmonary cryptococcosis, lymphoma, and invasive cervical carcinoma.

### Laboratory data

Whole blood (3.5 mL) was collected in serum separator gel tubes and centrifuged at 3500 rpm for 10 minutes, and serum was separated and processed for albumin, aspartate amino transferase (AST) and alanine amino transferase (ALT) using standard methods. Abnormal liver function testing was defined as an AST and/or an ALT value>35 mg/dl, the upper limit of normal in the testing laboratory. CD4 counts were determined with a FACS counter (Becton and Dickinson, Immunocytometry Systems, San Jose, CA, USA). Diagnosis of HIV infection was determined by having a positive result for HIV-1 antibodies ELISA kits (HIV Vironostika, Netherlands, and Murex HIV-1.2, Oxford, UK) which was confirmed by a positive result again with the same test.

### Statistical analysis

Separate analyses were done for participants with and without HIV infection. Further analyses were done for HIV positive participants by CD4 strata (>350,200–350 and <200 cell/µl). Descriptive statistics; (i.e mean, standard deviation), were used to present continuous variables, and number and percentages for categorical variables. Analysis of variance test for differences among continuous variables while Chi-square tests did so for categorical variables. Linear regression analyses assessed the relationships between the outcome variables (FMI and FFMI) and each independent variable. Stepwise selection regression was used to build multivariate models and a p-value of 0.10 was necessary to enter the model (p-values less than 0.05 were considered as statistically significant for other analyses). Potential confounding was examined by comparing coefficients of the same variables in univariate to those in multivariate models. Analyses were conducted using STATA 11.1 (StataCorp LP, College Station, TX, 2010).

## Results

Demographic, clinical and laboratory parameters of study participants are summarized in Table 1 by HIV/CD4 group (HIV−, HIV+-CD4>350, HIV+-200≤CD4≤350, HIV+-CD4<200). The HIV-negative women were older (P<0.001, for comparing all HIV/CD4 groups), more likely to be widowed, and less likely to live with a partner (p = 0.003,), and on average had larger family size (p<0.001). All clinical parameters (pulmonary tuberculosis, wasting and diarrhea in past 6 months) were significantly elevated in HIV positive women particularly those in lower CD4 strata (p<0.001 for all). Among the HIV positive women only, those with lower CD4 counts were more likely to report a prior stage IV WHO AIDS-defining condition (p<0.001). There was no significant difference by HIV/CD4 group in literacy, income, or having electricity in the home.

**Table 1:** Baseline characteristics of study participants (n = 895).

Participant Characteristics	HIV NEGATIVE (n = 217)	HIV POSITIVE (n = 678)			P-VALUE^a^
		CD4>350^b^ (n = 187)	CD4 200–350^b^ (n = 255)	CD4<200^b^ (n = 236)	
**Demographic parameters**					
Age, years (mean±SD)	42.4±10.5	34.2±6.9	35.2±6.7	34.9±7.1	<0.001
Marriage, n (%)					
Legally or living with a partner	77 (37.4%)	80 (43.2%)	94 (36.8%)	70 (30.0%)	0.003
Widowed	104 (50.5%)	68 (36.8%)	109 (42.7%)	103 (44.2%)	
Others	25 (12.1%)	37 (20.0%)	52 (20.4%)	60 (25.7%)	
Number of people live with you n (%)					
None,	0	7 (3.8%)	8 (3.2%)	16 (6.9%)	<0.001
Less than 3	22 (11.1%)	37 (20.1%)	59 (23.3%)	56 (24.2%)	
3–5 people	103 (52.0%)	98 (53.3%)	129 (50.9%)	114 (49.3%)	
More than 5	73 (36.9%)	42 (22.8%)	57 (22.5%)	45 (19.5%)	
Monthly Income, Rwandan Francs (US$)					
<10,000(18$)	90 (45.7%)	62 (33.7%)	95 (37.8%)	85 (37.1%)	0.055
10000–35000 (18–60$)	74 (37.6%)	101 (54.9%)	122 (48.6%)	109 (47.6%)	
>35 000 (>60$)	33 (16.7%)	21 (11.4%)	34 (13.5%)	35 (15.3%)	
Are Literate, n(%)	144 (69.9%)	141 (76.2%)	198 (77.9%)	177 (76.6%)	0.21
Have Electricity in house n(%)	21 (10.7%)	23 (12.50%)	36 (14.3%)	20 (8.7%)	0.26
**Clinical parameters**					
Diarrhea in past 6 months n(%)	17 (8.1%)	27 (14.6%)	56 (22.1%)	78 (33.5%)	<0.001
Pulmonary tuberculosis n(%)	4(1.9%)	19 (10.3%)	27 (10.6%)	41 (17.6%)	<0.001
Wasting n(%)	1 (0.5%)	12 (6.5%)	19 (7.5%)	33 (14.2%)	<0.001
WHO Class 4 n(%)	NA	45 (24.1%)	79 (30.9%)	89 (37.7%)	<0.001
**Anthropometric measurements**					
Body Mass Index ,kg/m^2^	21.3±3.8	21.9±3.9	21.9±3.9	21.1±3.7	0.09
Fat mass Index, kg/m^2^	3.9±3.1	4.1±3.1	4.5±3.3	4.1±3.2	0.30
Fat free mass Index, kg/m^2^	17.4±1.5	17.5±1.5	17.4±1.6	17.0±1.5	0.01
Sum of skin folds ,mm (Thigh, Triceps, Subscapular)	55±20	51.1±19.7	53.9±20.2	50.2±16.7	0.07
**Laboratory parameters**					
CD4, cells/ µl, (mean±SD)	NA	497±1.4	272±0.4	125±0.5	<0.001
Albumin g/dl, (mean±SD)	3.9±0.5	3.6±0.6	3.4±0.6	3.3±0.6	<0.001
Hemoglobin mg/dl (mean±SD)	14.3±1.4	13.5±1.2	13.1±1.7	12.7±1.7	<0.001
Abnormal Liver tests^c^, n(%)	12 (5.7%)	13 (7.4%)	19 (8.1%)	26 (11.8%)	0.13

a P-value is for comparing all four HIV/CD4 groups.

b n(%) for categorical variables or mean±standard-deviation for continuous variables.

c Alanine aminotransferase or aspartate aminotransferase>35 mg/dL.

While there was no significant difference in mean BMI or Fat Mass Index in the four HIV/CD4 groups (p = 0.09 and p = 0.3 respectively), there was a statistically significant difference in mean FFMI among the four groups: FFMI (kg/m^2^) was (mean standard-deviation) 17.4±1.5 in HIV negative and 17.5±1.5, 17.4±1.6, 17.0±1.5 in HIV positive women with CD4>350, 200–350 and CD4<200 cells/µl respectively (p = 0.01). Mean serum albumin (g/dl) systematically dropped from 3.9±0.5 in HIV negative to 3.6±0.6, 3.4±0.6 and 3.3±0.6 g/dl in HIV positive with CD4 count>350, 200–350 and <200 cells/ µl respectively (p<0.001). Among HIV negative women, 5.7% had abnormal AST or ALT (>35 mg/dl), which was systematically higher with HIV infection and lower CD4, at 7.4%, 8.1% and 11.8% of HIV positive with CD4 count<350, 200–350, and <200 cells/µl respectively, a difference that was not statistically significant by a standard chi-square test, but using a trend test obtained P = 0.024 (data not shown).

Univariate analyses of the associations of albumin and demographic, clinical and other laboratory variables with BMI, FMI and FFMI are presented in Table 2 stratified by HIV/CD4 group (HIV−, HIV+-CD4>350, HIV+-200≤CD4≤350, HIV+-CD4<200). Among demographic parameters, higher income (>10,000 compared to <10,000 Rwandan francs monthly was associated with higher BMI and Fat Mass Index in HIV negative women and in HIV positive with CD4>350, but not in the HIV+ women with CD4<350 cells/µl. Having electricity in their homes, a measure of “disposable” income, was highly associated with higher BMI and FMI among HIV negative women and in HIV positive groups with CD4>200. In HIV-negative and HIV positive women with CD4 count>350 cells/µl, serum albumin was not significantly associated with BMI, FFMI or FMI. However, In HIV+ women with CD4 count 200–350 cells/µl, higher albumin was significantly associated with higher BMI (1.027 kg/m^2^ per g/dl of albumin), FFMI (0.404 kg/m^2^ per g/dl of albumin) and FMI (0.655 kg/m^2^ per g/dl of albumin) (p = 0.006, p = 0.01, p = 0.04 respectively). These associations were even stronger in women with CD4 count<200 cells/µl: BMI (1.70 kg/m^2^ per g/dl increase in albumin), FFMI (0.73 kg/m^2^ per g/dl increase in albumin) and FMI (1.01 kg/m^2^ per g/dl increase in albumin) (P<0.001 for BMI and FFMI and 0.003 for FMI). Higher hemoglobin was highly associated with higher values for BMI, FFMI and FMI in HIV positive women with CD4<200 cells/µl (P<0.001).

**Table 2:** Univariate linear associations of albumin and other selected participant characteristics with body mass, fat mass and fat free mass index.

Participant Characteristics	Body Mass Index (kg/m^2^)				Fat Mass Index(kg/m^2^)				Fat Free Mass Index(kg/m^2^)			
	HIV-Negative	HIV-positive			HIV-Negative	HIV-positive			HIV-Negative	HIV-positive		
		CD4>350	CD4 200–350	CD4<200		CD4>350	CD4 200–350	CD4<200		CD4>350	CD4 200–350	CD4<200
	Regression co-efficient				Regression co-efficient				Regression co-efficient			
**Demographic parameters**												
Age (per 5 years)	−0.27^*^	−0.21	0.06	−0.24	−0.23*	−0.13	0.05	−0.18	−0.06	−0.04	0.02	0.004
Marital status												
Widowed vs. Married/Living with Partner	−0.88	−2.01*	−0.38	−0.002	−0.64	−1.41*	−0.12	0.10	−0.21	−0.53*	−0.40	0.06
Other vs. Married/Living with Partner	0.90	−0.62	−0.74	−0.59	1.08	−0.61	−0.43	−0.14	−0.12	−0.40	0.38	−0.28
Monthly Income, Rwandan Francs												
10000–35000 vs. <10000	1.43**	1.44*	1.19*	1.51**	1.28**	1.17*	0.98*	0.87	0.18	0.16	0.35	0.44
>35000 vs. <10000	2.88***	1.10	2.42**	1.45*	2.45***	1.33	1.65*	0.89	0.43	−0.02	1.06**	0.34
Number of People in household^1^	0.79*	0.42	0.28	−0.11	0.74*	0.48	0.08	−0.07	0.07	0.19	0.20	−0.11
Literate (Yes vs. No)	1.32^*^	0.59	0.66	1.53**	1.10*	0.29	0.45	0.73	0.09	0.05	0.52*	0.71**
Electricity in Home (Yes vs. No)	3.62***	2.03*	2.19**	0.64	3.14***	1.98**	1.68**	0.41	0.51	0.22	0.55	0.09
**Clinical parameters**												
Diarrhea in past 6 months	−0.62	−2.05*	−1.68**	−0.39	−1.23	−1.31	−1.03*	0.05	0.21	−0.44	−0.60*	−0.17
Pulmonary tuberculosis	0.60	0.30	−0.82	−0.79	1.23	1.34	−0.79	−0.92	−0.63	−0.21	−0.39	−0.02
WHO Class 4(excluding weight loss)	NA	−0.84	−1.01*	−0.37	NA	−0.63	−0.69	−0.07	NA	0.07	−0.38	−0.29
**Laboratory parameters**												
CD4 count (per 100 cells)	0.12	−0.25	0.40	0.75	0.07	−0.30	−0.40	0.41	0.06	0.08	0.44	0.15
Abnormal liver function tests (Yes vs. No)^2^	0.28	0.30	0.21	0.12	0.20	0.29	0.05	0.90	0.09	0.24	0.23	−0.48
Hemoglobin (mg/dl)	0.16	0.71**	0.18	0.60***	0.17	0.46*	0.19	0.40**	−0.03	0.16	0.05	0.27***
Albumin (g/dl)	−0.36	0.83	1.03**	1.70***	−0.16	0.77	0.65*	1.01**	−0.37	0.15	0.40*	0.73***

CD4 counts are cells/µl; Asterisks represent p-values for association within the column HIV/CD4 group (*<0.05, **<0.01, ***<0.001);

1 Categorized as none, 1–3, 3–5, >5, treated as continuous variable;

2 Aspartate amino transferase *or* alanine amino transferase >35 mg/dl.

We assessed albumin's independent association with BMI, FMI and FFMI by forward stepwise selection in multivariable linear regression models which adjusted for severity of HIV and other diseases. The only significant independent associations of serum albumin in these models was with FFMI in HIV+ women with CD4 counts 200–350 and <200 cells/µl: estimates 0.44 and 0.37 kg/m^2^ per g/dl of albumin, respectively, p = 0.03 for both. Albumin was not independently associated with FFMI in HIV negative women or HIV+ women with CD4>350 cells/µl (table 3). There was also no significant independent association of serum albumin with FMI in any of the groups (Table 4). In fact, albumin did not have a strong enough association to enter any models for FMI. However hemoglobin was independently associated with both FFMI (estimate in kg/m^2^ per mg/dl of hemoglobin, p-value) (0.19, p = 0.007) and FMI (0.4, p<0.02) in women with CD4<200 cells/µl.

**Table 3:** Final stepwise multivariate linear regression models for the association of serum Albumin and other selected characteristics with FAT FREE MASS INDEX (FFMI) among HIV-negative women and HIV Positive by CD4 strata^1^.

Participant Characteristics		HIV POSITIVE		
	HIV NEGATIVE	CD4>350	CD4 200–350	CD4<200
	estimate(95%CI) kg/m^2^	estimate(95%CI) kg/m^2^	estimate(95%CI) kg/m^2^	estimate(95%CI) kg/m^2^
Electricity (Yes vs. No)	0.48(−0.19, 1.63)			
Pulmonary tuberculosis (Yes vs. No)	−0.59(−2.1, 0.8)			
Marital status		−0.53*(−1.1, −0.01)		
Widowed vs. Married/Living with Partner		−0.40(−0.04, 0.2)		
Other vs. Married/Living with Partner				
Literate (Yes vs. No)			0.44(−0.06, 0.9)	0.53*(0.05, 1.01)
Diarrhea (Yes vs. No)			−0.55*(−1.05, −0.05)	
WHO stage IV			−0.22*(0.7, 0.2)	
CD4 count (per 100 cells)			0.41(−0.07, 0.9)	
Albumin (g/dl)			0.35*(0.04, 0.7)	0.41*(0.04, 0.7)
Hemoglobin (mg/dl)				0.19**(0.05, 0.3)

1 Numbers are kg/m^2^ changes in Fat Mass Index per unit increase in row covariate given all other variables in model are unchanged.

Asterisks represent p-values for association within the column HIV/CD4 group (*<0.05, **<0.01, ***<0.001).

**Table 4:** Final stepwise multivariate linear regression models for the association of serum albumin and other selected characteristics with FAT MASS INDEX (FMI) among HIV-negative women and HIV Positive by CD4 strata^1^.

Participant Characteristics		HIV POSITIVE		
	HIV NEGATIVE	CD4>350	CD4 200–350	CD4<200
	estimate(95%CI) kg/m^2^	estimate(95%CI) kg/m^2^	estimate(95%CI) kg/m^2^	estimate(95%CI) kg/m^2^
Electricity (Yes vs. No)	2.19**(0.7, 3.6)	2.38**(−6.9, 3.8)		
Monthly Income, Rwandan Francs				
10000–35000 vs. <10000	1.08*(0.1, 2.1)		0.86(−0.1, 1.8)	
>35000 vs. <10000	1.65**(0.3, 2.9)		1.57*(0.2, 2.9)	
Marital status				
Widowed vs. Married/Living with Partner	−0.05(−1.0, 0.9)			
Other vs. Married/Living with Partner	1.27(−0.2, 2.7 )			
Hemoglobin (mg/dl)		0.40*(0.002, 0.8)		0.40**(0.14, 0.6)
WHO stage IV			−0.70(−1.7, 0.26)	

1 Numbers are kg/m^2^ changes in Fat Mass Index per unit increase in row covariate given all other variables in model are unchanged.

Asterisks represent p-values for association within the column HIV/CD4 group (*<0.05, **<0.01, ***<0.001).

## Discussion

Serum albumin level is commonly used as a nutritional marker in many clinical settings [14], [15], [26] because of its ease of measurement and its inclusion in many chemistry profiles. However, our study found that serum albumin did not predict other measures of nutritional status (BMI, FFMI or FMI) in either HIV-negative or positive African women, with the exception of one small subgroup. The only significant associations of serum albumin with the body composition indices we measured was with FFMI in HIV+ women with very advanced disease (CD4<200cells/µl), suggesting that the lower serum albumin level was a marker of the catabolic state of later-stage disease, rather than of poor nutritional status. For example, in patients found to be severely malnourished on clinical examination with BMI<18.5 the mean serum albumin was 4.0 g/dl in HIV negative and 3.1 g/dl in HIV positive women (data not shown). Thus, despite their low BMI, the HIV negative women tended to have serum albumin levels in the normal range of 3.4 to 5.4 g/dl whereas in HIV positive women with BMI<18.5 kg/m^2^ the mean serum albumin level was well below the lower limit of this range. This suggests a specific effect of advanced HIV disease in lowering BMI and serum albumin rather than a direct association of low serum albumin with low BMI.

Albumin levels are influenced by many clinical manifestations other than nutritional status [14] , which may limit the utility of using serum albumin as a specific marker of malnutrition. For example, illnesses that increase albumin loss, such as nephrotic syndrome, or diseases that decrease albumin synthesis, such as cirrhosis, will result in lower albumin levels regardless of nutritional status [27]. To that end, we found that lower values of serum albumin correlate with lower CD4 cell count. The mean serum albumin systematically dropped from 3.9±0.5 g/dl in HIV negative to 3.6±0.6, 3.4±0.6 and 3.3±0.6 g/dl in HIV positive participants with CD4 count>350, 200–350 and <200 cells/µl respectively. Our findings are similar to the results from a Kenyan study which indicated that a decrease in albumin of over 10% was associated with a 3.5-fold increase in the risk of progressing to a CD4 count<200 cells/µl [28]. Feldman and colleagues in their study of serum albumin as a predictor of survival in HIV-infected women in the Women's Interagency HIV Study, reported that a single measurement of albumin at baseline was very prognostic of survival in patients with CD4 cell counts of <200 cells/µl, with the hazard of death increasing 8-fold in those with albumin <35 mg/L compared with those with albumin >45 mg/l [19].

Prior studies of US patients have reported that even early-stage of HIV infection results in considerable loss of body weight and changes in body fat distribution [29]. In contrast to these studies, we found that among ART naïve HIV-infected Rwandan women the BMI, body fat index, and fat-free mass index (among those with CD4>200 cells/µl), were similar to those found in HIV-uninfected women (table 1). Our findings are supported by a prior study conducted in South Africa assessing body composition of South African lactating women in relation to HIV serostatus which demonstrated no significant difference in mean BMI (24.6 and 25.3 kg/m^2^ with a p-value of 0.3) in HIV-infected and uninfected women, respectively [30].

In this study, we did not use a gold standard measurement of body composition, such as the dual-energy x-ray absorptiometry. Furthermore, the BIA prediction method used has not yet been validated in the Rwandan population. As a result, our estimates of body composition may not be accurate because of variations across ethnic groups [21]; this would make it harder to detect any associations that existed between albumin and the different body composition components. However, we believe any such effect would be small as we have used gender specific equations that were previously cross validated in individuals of different race groups (white, black, and Hispanic) among men and women, who were both healthy controls and HIV-infected patients [23]. Moreover, the equations have been used widely in other studies from Africa with meaningful findings [24], [25], [31]. Our study is also limited in determination of direction of causality by its cross-sectional design and in generalizability to men as the study population consisting of only women. However, as 60% of HIV-infected Africans are women [32] and body composition is different in women compared to men, it is important to assess metabolic parameters in African women specifically.

In summary, we have found that malnutrition, defined by the WHO standard of low BMI, is common in Rwandan women, independent of HIV status, but that no measure of nutritional status in our cohort was systematically associated with serum albumin. Our study suggests that albumin should not be used as a proxy for nutritional status in HIV infected Rwandan women and probably in other individuals with advanced disease in both developing and developed countries. Further studies of association of serum albumin with validated measures of nutritional status are needed in diverse populations. Other specific markers such as transthyretin (also known as pre-albumin) that have shown to be sensitive in assessing nutritional status [33] should also be validated in a similar fashion.
